# Supplementary material for: Identification of photoperiod-induced specific miRNAs in the adrenal glands of Sunite sheep (Ovis aries)
Source: Front Vet Sci. 2022 Jul 22;9:888207. doi: 10.3389/fvets.2022.888207 (PMC9354845; doi:10.3389/fvets.2022.888207)
Supplement: Supplementary file 1 [file Data_Sheet_1.ZIP › Supplementary file/miRDeep2 file.zip!/miRDeep2 file/Novel_Structure/SP42A2/Novel_228.pdf]

Provisional ID	:	NC_019484.2_43040
Score total	:	50.6
Score for star read(s)	:	3.9
Score for read counts	:	43
Score for mfe	:	2.2
Score for randfold	:	1.6
Score for cons. seed	:	
Total read count	:	96
Mature read count	:	95
Loop read count	:	0
Star read count	:	1

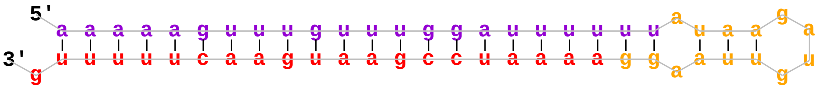

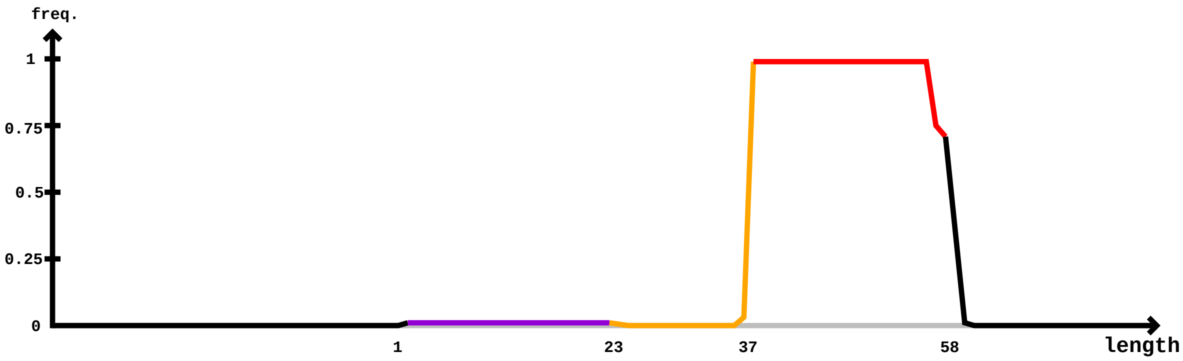

Star Mature

```
5'- uacaugugcgaauuguuacuaauauugggcuggccaaaaaguuuguuuggauuuuuuauaagauguuaaggaaaauccgaaugaacuuuuuggccugcccaauacaucugcca   -3'   obs
    uacaugugcgaauuguuacuaauauugggcuggccaaaaaguuuguuuggauuuuuuauaagauguuaaggaaaauccgaaugaacuuuuuggccugcccaauacaucugcca         exp
    ......................((((((((.((((((((((((((((((((((((((.(((....))).)))))))))))))))))))))))))).)))))))).........   reads   mm   sample
    ...................................aaaaaguuuguuuggauuuuuu........................................................   1       0    seq
    ......................................................................gaaaauccgaaugaacuuuuug.....................   1       0    seq
    ......................................................................gaaaauccgaaugaacuuuuugg....................   2       0    seq
    .......................................................................aaaaucUgaaugaacuuuu.......................   23      1    seq
    .......................................................................aaaauccgaaugaacuuuuu......................   4       0    seq
    .......................................................................aaaaucAgaaugaacuuuuug.....................   1       1    seq
    .......................................................................aaaaucUgaaugaacuuuuug.....................   57      1    seq
    .......................................................................aaaauccgaaugaacuuuuug.....................   3       0    seq
    .......................................................................aaaauccgaaugaacuuuuugg....................   3       0    seq
    .......................................................................aaaauccgaaugaacuuuuuggc...................   1       0    seq
```
